# Investigating the effective factors of near-drowning experience in junior high school students of Guilan Province, Iran: a theory-based study 

**Published:** 2019-08

**Authors:** Seyedeh-Tahereh Nopasand-Asil, Leili Tapak, Akram Karimi-Shahanjarini, Forouzan Rezapur-Shahkolai

**Affiliations:** ^*a*^Hamadan University of Medical Sciences, Hamadan, Iran.

## Abstract

**Background:**

Drowning is one of the most important causes of deaths from injuries in the world, and among adolescents. Although drowning is one of the major public health threats, it can be prevented by knowing its effective factors. Purposes: This study aims to investigate the effective factors of near-drowning in junior high school students, based on Theory of Planned Behavior.

**Methods:**

This cross-sectional study was conducted in 2018 in Guilan province, north of Iran. Of the seven coastal cities, three cities were randomly selected as samples. The data gathering tool was a researcher-made questionnaire including questions about demographic variables, knowledge and constructs of the Theory of Planned Behavior (attitude, subjective norms, perceived behavioral control, intention and behavior) and near-drowning. After confirming the validity and reliability, students completed the questionnaire. 734 students were randomly selected using multi-stage cluster sampling. Data were analyzed using logistic regression, linear regression and SPSS-16 software.

**Results:**

21.1% of the students had near-drowning experience. 59.4% of the injuries were occurred in the sea and the rest of them were in pool, river, dam, pond, bathtub, well and canal. Most of the injuries were occurred at 4-8p.m (35.5%). Near-drowning following swimming (41.3%), playing and recreation (23.9%), and diving (12.3%) were the most common mechanisms. Logistic regression showed a significance between sex (p = 0.000), father s education (p = 0.005), place of residence (p = 0.000), swimming skills (p = 0.003), intention (p = 0.020) and near-drowning. Also linear regression revealed a significant relationship between attitude and drowning prevention behaviors (p = 0.018).

**Conclusions:**

In the studied population, the near-drowning rate is high. Considering most of the injuries occurred in the sea following swimming, accurate knowledge of all effective factors of near-drowning will help design appropriate interventions to prevent drowning in adolescents, including beach safety, setting time for swimming in the sea and swim training.

**Keywords:**

Drowning; Injury Prevention; Safety promotion; Health promotion

